# Leaf-Movement-Based Growth Prediction Model Using Optical Flow Analysis and Machine Learning in Plant Factory

**DOI:** 10.3389/fpls.2019.00227

**Published:** 2019-03-22

**Authors:** Shogo Nagano, Shogo Moriyuki, Kazumasa Wakamori, Hiroshi Mineno, Hirokazu Fukuda

**Affiliations:** ^1^Department of Mechanical Engineering, Graduate School of Engineering, Osaka Prefecture University, Sakai, Japan; ^2^Japan Society for the Promotion of Science, Tokyo, Japan; ^3^Graduate School of Integrated Science and Technology, Shizuoka University, Shizuoka, Japan; ^4^PRESTO, Japan Science and Technology Agency, Kawaguchi, Japan

**Keywords:** circadian clock, lettuce, machine learning, optical flow, phenotyping, plant factory

## Abstract

Productivity stabilization is a critical issue facing plant factories. As such, researchers have been investigating growth prediction with the overall goal of improving productivity. The projected area of a plant (PA) is usually used for growth prediction, by which the growth of a plant is estimated by observing the overall approximate movement of the plant. To overcome this problem, this study focused on the time-series movement of plant leaves, using optical flow (OF) analysis to acquire this information for a lettuce. OF analysis is an image processing method that extracts the difference between two consecutive frames caused by the movement of the subject. Experiments were carried out at a commercial large-scale plant factory. By using a microcomputer with a camera module placed above the lettuce seedlings, images of 338 seedlings were taken every 20 min over 9 days (from the 6th to the 15th day after sowing). Then, the features of the leaf movement were extracted from the image by calculating the normal-vector in the OF analysis, and these features were applied to machine learning to predict the fresh weight of the lettuce at harvest time (38 days after sowing). The growth prediction model using the features extracted from the OF analysis was found to perform well with a correlation ratio of 0.743. Furthermore, this study also considered a phenotyping system that was capable of automatically analyzing a plant image, which would allow this growth prediction model to be widely used in commercial plant factories.

## Introduction

Closed-type plant factories, which cultivate plants in closed systems with controlled temperature, humidity, and light, are attracting attention as a new type of cultivation method, capable of producing the extra food needed to respond to population growth, while protecting the environment, improving health, and achieving economic growth (Kozai et al., 2015; Anpo et al., 2018; Kozai, 2018). However, these closed-type plant factories are more costly than outdoor cultivation because of the initial costs and the running costs incurred in the control of the environment. To reduce these costs, many studies have been undertaken, addressing the effect of light quality on plant growth and quality (Tamura et al., 2018), and the optimization of the air flow (Takahashi et al., 2012).

Plant growth prediction is one solution to overcoming these problems. Poorly grown plants that do not satisfy the level of quality required for sale leads to serious losses (Kozai et al., 2015). Poor growth of the plants occurs due to individual differences, even when the seeds are cultivated under the same conditions. Thus, to make plant factories viable, seedling diagnosis technology is an important concept. Such technology should mainly use visual information from plants, recognize the differences between individual plants, and then identify and cull low-grade plants at an early stage (Fukuda et al., 2011; Moriyuki and Fukuda, 2016; Moriyuki et al., 2018). In a previous study, the authors’ group constructed a high-throughput growth prediction model for lettuce cultivars based on chlorophyll fluorescence for application to a commercial plant factory (Moriyuki and Fukuda, 2016). Furthermore, this prediction model uses the circadian rhythm extracted from the chlorophyll fluorescence, because circadian rhythm is responsible for regulating growth (Dodd et al., 2005; Harmer, 2009). In addition, there are individual differences in the circadian rhythm of lettuce cultivars (Ukai et al., 2012; Higashi et al., 2014), and the growth rate of lettuce plants depends on circadian rhythms, which entrain to various period of light-dark cycles (Higashi et al., 2015). From these points of view, it is speculated that the measurement of circadian rhythms will lead to an improved accuracy of plant growth prediction, leading the authors to focus on the relationship between the circadian rhythms and the visual information of the plants.

Leaf movement is recognized as being an important visual information that is related to the circadian rhythm (Halaban, 1969). The relationship between leaf movement and circadian rhythms has been researched on the laboratory scale (Edwards and Millar, 2007), but few commercial large-scale experiments have been attempted. To precisely extract the circadian rhythms, this study focused on OF analysis. OF analysis is an image-processing method that recognizes and extracts the difference between two consecutive frames caused by the movement of the subject of the image. In addition to the computer-vision field, OF analysis is used in the field of plant science, in applications such as the recognition of water stress in tomato vines (Kaneda et al., 2017). In addition, the importance of the relation between the angle and plants has been reported by Okabe (2015), who used a mathematical model to determine that the golden angle of phyllotaxis, which is defined by the twisting of the stem is a key factor for minimizing energy costs. To precisely extract plant growth and angle information, the authors propose the application of normal-vector analysis. Normal-vector analysis is a post-processing method applied after OF analysis, which converts every vector calculated by OF analysis to a normal vector, relative to the center of the seedling. By using normal-vector analysis, the plant growth, stagnation of the plant growth, and the direction of the leaf extension can be determined. In this study, a machine learning model is used, which employs these features to predict the growth of plants.

Machine learning is a promising technique for the analysis of large amounts of data and is mostly performed for prediction and classification tasks. This method is widely used in various research fields, including plant production, plant science, and plant phenotyping (Moriyuki and Fukuda, 2016; Singh et al., 2016; Gutiérrez et al., 2018; Moghimi et al., 2018; Pineda et al., 2018; Zhang et al., 2018). In this study, gradient boost regression (GBR) was selected as the prediction model (Friedman, 2001). GBR is a machine learning technique for regression, which produces a prediction model in the form of an ensemble of weak prediction models, called a “decision tree.” It builds a model, stage by stage, and then generalizes the model by allowing the optimization of an arbitrary differentiable loss function. This algorithm is also able to visualize the feature importance, enabling not only growth prediction but also the identification of the contributions of features derived from normal-vector analysis.

This study involved extraction of the image data features that are related to the leaf movement and the subsequent use of machine learning to construct a growth prediction model for lettuce, which is a typical crop grown in a closed-type plant factory. Using machine learning, the authors attempted to predict the final fresh weight at harvesting using 22 days before the seedling diagnosis data was collected. The experiments were performed in an actual commercial large-scale plant factory with a daily output of 5,000 lettuces.

## Materials and Methods

**Figure 1:**
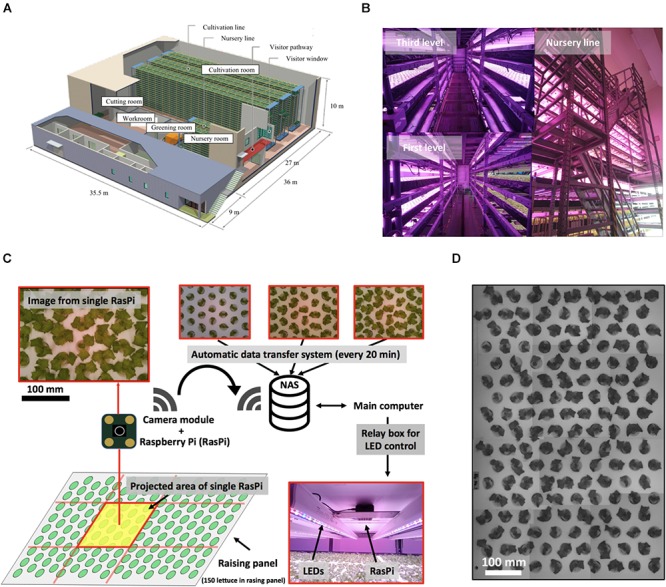
Multiple plant imaging (MPI) system in commercial plant factory. **(A)** Production line of commercial plant factory in Osaka Prefecture University. Successive operations, including greening, nursing, cultivation, and cutting, are performed. **(B)** Nursing room. This room has three levels (left-hand upper figure shows the third level. The lower figure shows the first level). This nursery line (right-hand figure) has a capacity to accommodate 10^5^ seedlings. **(C)** MPI system for acquiring feature values for each seedling based on time-series photographs captured on the nursing line. **(D)** Simultaneous measurement of seedlings on nursing panel using MPI system.

This study was performed in a commercial large-scale plant factory (Figure 1A). The plant growth occurred in three stages, namely, the greening, nursery, and cultivation stages. The acquisition of plant images was carried out in the nursery stage, while the fresh weight of the plants was measured upon harvesting at the end of the cultivation stage.

### Plant Material and Growth Conditions

Experiments were carried out using lettuce seeds (*Lactuca sativa* L. cv. SB555GL, a fixed line of lettuce cultivar offered by Snow Brand Seed Co., Sapporo, Japan). First, in the greening stage, each plant was seeded in a greening panel (a urethane sponge carrying 600 plants) together with 5 L of tap water and fertilizer (OAT house, OAT Agrio Co., Ltd, Tokyo, Japan). Second, the greening panel was placed in a dark growth chamber at 25°C for 2 days to allow the seeds to germinate. Third, the plants were cultivated for 4 days under white LED light (LIFELED’S; NEC Lighting, Ltd., Tokyo, Japan) at a light/dark ratio of 15/9 h. Fourth, the plants were cultivated in a nursery panel for 14 days under LED light [blue, white, red, and far-red LEDs (GreenPower LED production module DR/W/FR 120; Philips, Amsterdam, Netherlands], again at a light/dark ratio of 15/9 h at pH 6.0, EC 0.12 S m^-1^, and 23.5 ± 1.0°C and 21.5 ± 1.2°C in the light and dark periods, respectively (Figure 1B). The acquisition of an image by the proposed multi-plant imaging system was performed from above the nursery panel every 20 min. Finally, in the cultivation stage, 150 lettuces per nursery panel were moved to the cultivation panel for one experiment, where they were cultivated for 18 days and then harvested. The temperature of the cultivation panel was 23.5 ± 1.0°C and 21.5 ± 1.2°C in the light and dark periods, respectively. The fresh weight of the aerial part of each lettuce was measured at 38 days after sowing. The experiment was performed three times.

### Automatic Plant Measuring System

The proposed multiple plant imaging (MPI) system (Figure 1C) was configured above the nursery panel in the nursery room. The MPI system captured image data of the projected area (PA) of seedlings using an accessible, low-cost microcomputer (RasPi; Raspberry Pi 3; Raspberry Pi Foundation, Cambridge, United Kingdom) and a camera module (Raspberry Pi Camera V2; Raspberry Pi Foundation, United Kingdom) to perform high-throughput phenotyping (Minervini et al., 2015; Tovar et al., 2018). Twelve RasPi units were used to allow the capturing of an image of the entire nursery panel (Figure 1D). These data were transferred automatically to network-attached storage (NAS, LinkStation LS520 Series; BUFFALO INC., Aichi, Japan) which could be accessed from a main computer. During the dark phases of the raising stage, images were captured by controlling the LEDs with a relay box, with the lights being turned on/off under the control of a signal from the main computer. The resolution of each image was 3280 × 2646 pixels. A total of 640 images (image dataset) were captured by each RasPi from 6 to 15 days after sowing, with a total of 18,560 images being used in the present study. The time point 6 days after sowing was defined as *t* = 0 h, such that the dataset covered the period from *t* = 0–201 h.

### Extraction of Leaf Movement From MPI System

**Figure 2:**
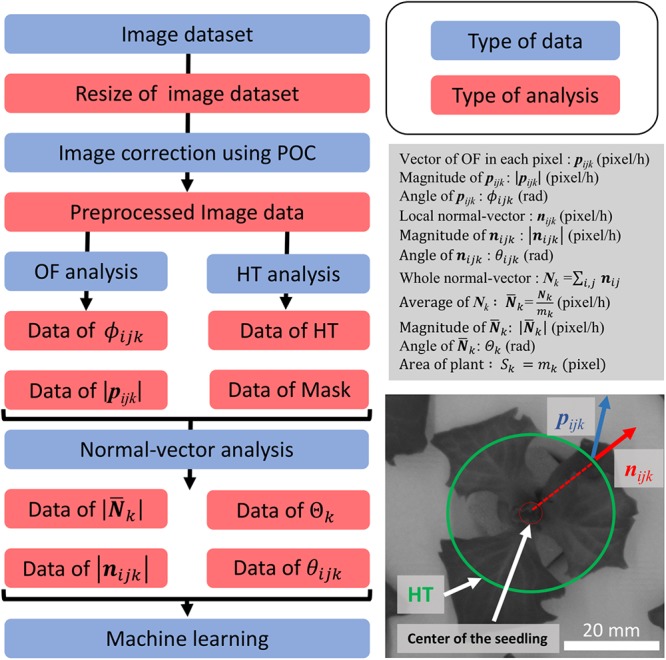
Summary of analyses performed, and conceptual drawing of normal-vector analysis. The flowchart on the left shows the preprocessing process for the dataset. The process with the blue background shows the type of data which were focused, and the red background shows the type of analysis method used for preprocessing. The description in the gray box shows the definition used in this study. The image on the bottom right shows the concept of our study. The green circle shows the detection of the panel using HT. The blue vector is the OF vector, and the red circle shows the local-normal vector.

Using the image dataset obtained with the MPI system, an OF analysis was programmed in Python 2.7.13 and OpenCV 3.1.0, while normal-vector analysis was programmed in Python 3.6.5 and OpenCV 3.1.0. A modeling diagram is shown in Figure 2. The image dataset was analyzed as follows. First, the images were resized from 3280 × 2464 to 640 × 480 pixels. The resizing algorithm was based on bilinear interpolation. Next, phase-only correlation (POC) was performed to correlate the small movement of the panel between the image dataset caused by its floating. Resizing and POC were performed on all the images of the dataset. For the OF analysis, the “DeepFlow” algorithm was used (Weinzaepfel et al., 2013). The DeepFlow algorithm was applied using two sets of images, captured 12 h apart. The images were resized to 320 × 240 pixels, and the OF was analyzed for each pixel. The OF vector in each pixel was defined as ***p***_ijk_, where *i* is the width of a pixel (1 ≤ *i* ≤ 320), *j* is the height of a pixel (1 ≤ *j* ≤ 240), and *k* is the number of the individual image being analyzed (37 ≤ *k* ≤ 640). After the calculation of the ***p***_ijk_, the ***p***_ijk_ of the seedling was separated and extracted from that of the background of panel by using an image binarization method called Excess Green (ExG; Reid et al., 2016). ExG was applied using the resized image, with the ExG threshold set to 0.2. Further, a hough transformation (HT), which is an object detection method, was used to detect the circular shape of the depression in the panel (Ballard, 1981). In this work, the center of this circle, as determined using HT, was defined as the center of the seedling, and the circle consisted of 28 pixels, with a radius of 2.5 cm from the center of the seedling. Additionally, a masked area was defined, namely, the area outside of the circle.

The local normal vector ***n***_ijk_ was calculated for every pixel using ***p***_ijk_ and the center of the seedling. The overall normal vector (***N***_k_) and average of ***N***_k_ (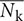) were defined as follows:

$$N_{\text{k}}\;=\;\sum_{\text{i},\text{j}}n_{\text{ijk}}$$

$$\bar{N_{\text{k}}}\;=\;\frac{N_{\text{k}}}{m_{\text{k}}}$$

where m*_k_* is the total number of ***n***_ijk_ The magnitude of 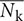 was defined as 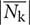 and the angle of 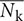 was defined as θ_k_. The projected area of the plant (PA) was defined as follows:

$$S_{\text{k}}\;=\;m_{\text{k}}$$

### Dimension Reduction for Machine Learning

To develop a prediction model based on the results of the normal-vector analysis, dimension reduction was applied, owing to the large size of the dataset. The dimensions of the dataset were reduced from 1,812 dimensions (604 time-series data points for the angle, magnitude, and PA) to 115 dimensions, as shown in Table 1. In this study, the authors chose two different ways to reduce the number of dimensions. The first way was a time-series analysis, as shown in Table 1. This dimension reduction method was used to extract the time-indicated features from the full-dimensional dataset. The second way was a principal component analysis (PCA), which is also shown in Table 1. The dimensions were reduced to increase the accuracy of machine learning. The prediction without the dimension reduction (i.e., using 1,812 dimensions) resulted in a correlation coefficient of 0.599 using a support vector regression (SVR). Feature “PPFD” is the photosynthetic photon flux density (ppfd) data, measured while the light is on for each area of the panel. Feature “Track” is that area in the panel which exhibits a difference in the interaction between each plant (Supplementary Figure S1). Feature “PCA” shows the result of dimension reduction using principal component analysis (pca) for all the dataset, including the angle data, magnitude data, and PA data. The features “angle-shift” and “magnitude-shift” indicate the correlation between the original time-series data and shift data of the angle and magnitude data, respectively. Feature “PA-ave” is the average of the PA data calculated every 24 h. Features “angle-diff,” “magnitude-diff,” and “PA-diff” are the data used to calculate the difference between two different time points for the angle, magnitude, and PA, respectively.

**Table 1:** Dimension reduction process.

		Number of
Type	Features	features	Descriptions of features
Environment	PPFD	1	Photosynthetic photon flux density (ppfd) data, measured while the light is on for each area of the panel
	Track	4	Area in the panel which exhibits a difference in the interaction between each plant
Dimension reduction	PCA	20	Principal component analysis (pca) with *n* component of 5 for all the dataset, including the angle data, magnitude data, and PA data
Time-series analysis	Angle-shift	6	Correlation between the original time-series data and shift data of the angle data
	Magnitude-shift	6	Correlation between the original time-series data and shift data of the magnitude data
	PA-ave	9	Average of the PA data calculated every 24 h
	Angle-diff	23	The data used to calculate the difference between two different time points for the angle
	Magnitude-diff	23	The data used to calculate the difference between two different time points for the magnitude
	PA-diff	23	The data used to calculate the difference between two different time points for the PA
Total number of features		115	

### Growth Prediction Model Using Machine Learning

For machine learning modeling, GBR, and SVR were performed. GBR is a type of ensemble learning combining multiple weak learners in order to overcome the overfitting of the model. The hyperparameters of GBR were set to min_samples_split: [10, 30, 50, 70], max_depth: [4, 6, 8, 10], subsample: [0.7–1], and learning_rate: [0.01, 0.05, 0.1]. Here, min_samples_split is the minimum number of samples required to split an internal node. The greater the value is, the more the overfitting of the parameter is reduced. In addition, max_depth is the parameter for the maximum depth of the individual regression estimators. A The greater the value is, the more complex are the features that the models describes. However, a high value might result in overfitting the training dataset. The parameter learning_rate shrinks the contribution of each tree by the value of learning_rate. The parameter subsample is the fraction of samples to be used for fitting the individual base learners. Choosing subsample <1.0 reduces variance and increases bias. The range and resolution of the parameters were set roughly based on the default parameters of the scikit-learn package and optimized by using grid search methods, which compare all combinations. SVR involves the use of algorithms based on kernels that transform the original data into high-dimensional feature spaces (Capparuccia et al., 1995). Before applying SVR, the dataset was normalized using the MinMaxScaler function of the scikit-learn package. The hyperparameters of SVR were set to C: 2^a^ (*a* = -20, -19, -18, … 18, 19, 20), *γ*: 2^b^ (*b* = -20, -19, -18, … 18, 19, 20), and 𝜀: 2^c^ (*c* = -20, -19, -18, … 18, 19, 20), respectively, while the radial basis function (RBF) was chosen as the kernel. The range and resolution of parameters were set over a wide range in this study because the computational cost was low in the dataset of this study and computing environment. The limitation of the grid range will be an important factor for a larger dataset, which must be focused on in the future. Before selecting a hyperparameter, the training and test data were divided randomly into 236 and 102 items of data, respectively (train:test = 7:3). The hyperparameters were selected using a stratified five-fold cross validation. Cross validation is a method used for machine learning in order to avoid the phenomenon called “overfit,” which is a modeling error in which the model is adjusted too specifically to a given dataset. In a five-fold cross validation, five models are validated with four folds and are then tested with the remaining fold five times, using different combinations. In the present study, five-fold cross-validation was carried out using random-fold splits. The coefficient of determination (*R*^2^) was used for the hyper-parameter tuning, while the correlation coefficient was used to visualize and evaluate the models. The model was described for GBR using the feature importance method. These models were developed using Python 3.6.5 and scikit-learn 0.19.1.

## Results

### Visualization of Optical Flow and Normal Vector

**Figure 3:**
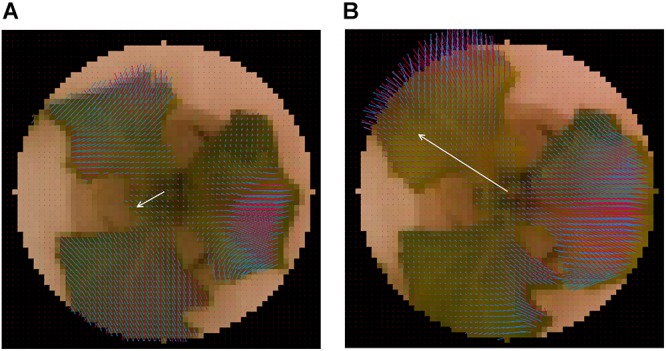
Visualization result of normal-vector analysis. Visualization result for single lettuce at **(A)** *t* = 168 h and **(B)** *t* = 180 h. Blue vector represents ***p***_ijk_ Red vector represents ***n***_ijk_. White vector represents 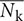. The visualization of 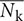 was performed by at 20 × the original 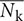.

Figure 3 shows the visualization results for ***p***_ijk_, ***n***_ijk_, and 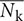 for a single lettuce. Figure 3A,B shows the visualization result for a single lettuce at *t* = 168 and 180 h, respectively. The value of ***p***_ijk_ was determined using an image captured 12 h before the plants were visualized (e.g., the images captured at *t* = 156 and 168 h are used to calculate the result shown in Figure 3A). The black area in the corner of Figure 3 is the masked area. To extract the vector of the growth direction, the values of ***n***_ijk_ were calculated using ***p***_ijk_ and the centers of the plants. By focusing on ***n***_ijk_ and 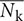, the growth of three leaves of the plant was found to be relatively uniform while the light was off (Figure 3A), with deviations occurring only when the light was on (Figure 3B). In addition, the angle of ***n***_ijk_ (θ_ijk_) can be divided into two categories, namely, the inward and outward directions. The inward directions represent the mixed information between the leaf-extending and leaf-drooping while the outward directions represent leaf-standing.

### Time-Series Analysis of Single Lettuce

**Figure 4:**
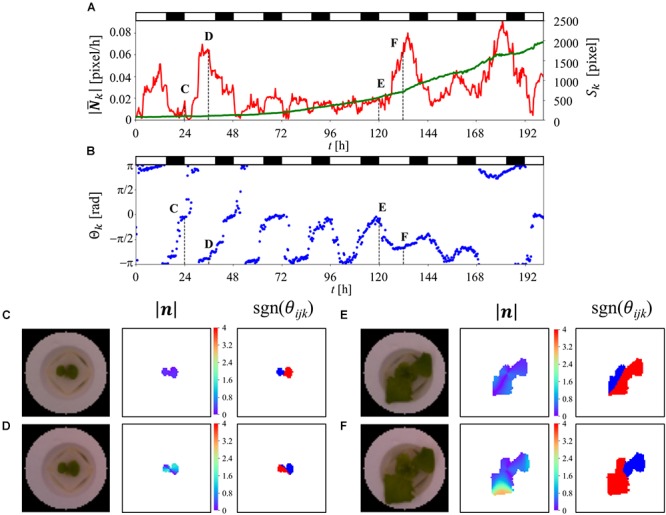
Time-series analysis result for single plant using OF. **(A,B)** Time-series analysis result for single lettuce from *t* = 0–201 h. Red, green, and blue lines represent 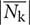, *S*_k_, and θ_k_, respectively. The white and black bar at the top of the figure indicates the light and dark conditions. **(C–F)** shows the image data and visualization results for |***n***_ijk_| as well as the visualization results for sgn(θ_ijk_) at *t* = 24, 36, 120, and 132 h, respectively. The red part of sgn(θ_ijk_) represents the positive-direction vector, relative to the center of the seedling. The blue part of sgn(θ_ijk_) represents the negative-direction vector, relative to the center of the seedling.

Figure 4A,B shows the time-series of 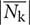, *S*_k_, and θ_k_ for a single plant. Based on these results, it was found that 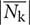 and θ_k_ exhibit a periodicity that is associated with the LD cycle. The result obtained for 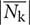 contains some noise, especially prior to *t* = 120 h. In addition, the results obtained for θ_k_ represent the different patterns obtained before and after *t* = 48 h. The plot of θ_k_ prior to *t* = 48 h shows stagnation of data points near -π, 0, and π, which correspond to the direction of the leaves in this stage, where the plot of θ_k_ after *t* = 48 h is in the range of –π to 0 rad, which indicates a direction that does not match the direction of the leaf at this stage. This is due to the composition of the two different directions of the leaf, where one is oriented to approximately π/4 rad and the other is oriented to approximately 5π/4 rad. *S*_k_ exhibits an exponential growth, which is in good agreement with the result reported by Evans (1972). Figure 4C–F show a visualization of image, |***n***_ijk_| and sgn(θ_ijk_) at *t* = 24, 36, 120, and 132 h, respectively. From these results, it was found that |***n***_ijk_| increases over time, while there is a time point at which the growth of the leaves is relatively equal, as shown in Figure 4C,E, while the distribution has a large deviation, as shown in Figure 4D,F. In Figure 4C–F, sgn(θ_ijk_) indicates that both the inward and outward directions of ***n***_ijk_ are observed in a single plant, with the corresponding area reversing in 12 h. A comparison of the distribution of sgn(θ_ijk_) in Figure 4E,F reveal that Figure 4F exhibits an imbalanced distribution of the inward and outward directions, which results in a high value of 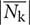, as shown in Figure 4A.

### Growth Prediction Using Machine Learning

**Figure 5:**
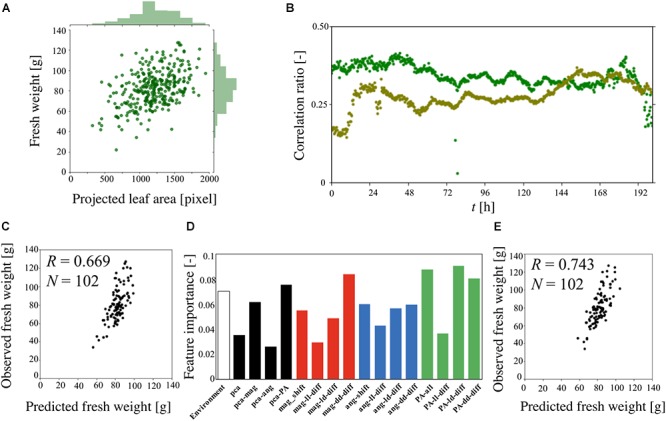
Predicted fresh weight using machine-learning method. **(A)** Correlation ratio between fresh weight at the harvest and PA at *t* = 180 h. **(B)** Correlation ratio between the fresh weight and PA from *t* = 0–201 h. The dark green and light green indicate the different sets of experiments (dark green: 141 plants, light green: 92 plants). **(C)** Correlation ratio between observed fresh weight and predicted fresh weight using gradient boost regression (GBR). **(D)** Feature importance analysis of 115 features using GBR. **(E)** Correlation ratio between observed fresh weight and predicted fresh weight using support vector regression (SVR).

Figure 5A is a scatter plot of the fresh weight at the harvest and PA at *t* = 180 h, combined with histograms of the data extracted from three different experiments (total of 338 plants). The histograms exhibit Gaussian distributions based on the Kolmogorov-Smirnov test. The calculated coefficient correlation was 0.454. Figure 5B shows the changes in the correlation ratio between the fresh weight at the harvest and each value of PA at *t* = 0–201 h. This result indicates that the correlation ratio of a single experiment is lower than the result shown in Figure 5A. Furthermore, the time-series result obtained for two different experiments revealed a different pattern, highlighting the difficulty of predicting the fresh weight when using only once measuring of PA.

Machine learning was applied to predict the fresh weight from the extracted large dataset that was constructed from Table 1. Three types of features and a total of 10 features and 115 dimensions were used for the machine learning. The features of the “PPFD” and the “Track” were made for the environmental features. The “Track” feature indicates the area of the panel, as shown in Supplementary Figure S1, which represents the interaction between the plants. In particular, in track 1, there is less interaction with the other plants than there is in tracks 2, 3, 4, and 5. To compare the importance of the features obtained with the statistical methods and circadian-related methods, “PCA” was used as a statistical means of dimension reduction. “PCA” was performed in four different ways, all of which used all the angle data, magnitude data, and PA data. For the time-series analysis of the features, six features were examined. The features “angle-shift” and “magnitude-shift” were calculated by determining the correlation between the original time-series data and the data which are shifted by 24, 48, 72, 96, 120, and 144 h for the angle data and magnitude data, respectively. This approach considers the periodicity of the time-series data, which is an important characteristic of circadian rhythms. The average value of PA is determined by averaging the PA for 0–24, 24–48, 48–72, 72–96, 96–120, 120–144, 144–168, 168–192, and 192–201 h. This simple approach was conducted for PA because the original time-series data for PA exhibits a relatively simple trend. For the “angle-diff” and “magnitude-diff” features, the averages of each of the light and dark conditions (LD) were calculated for the angle data and magnitude data, respectively. Then, the combination of the absolute difference between two consecutive light conditions (LL), dark conditions (DD), and pairs of LD was calculated. This approach to dimension reduction represents the effect of the circadian rhythms derived from the change in the light conditions, which is also known to be an important characteristic of a circadian rhythm.

Gradient boost regression is used for both the prediction of the fresh weight and to explain the importance of each feature. SVR was performed to increase the accuracy of the prediction, for which there are no methods for explaining the importance of features such as GBR. Figure 5C is a scatter plot of the observed and predicted fresh weight using GBR. Using this model, the correlation ratio was found to be 0.669. Figure 5D shows the feature importance as calculated from the result of GBR shown in Figure 5C. The white, black, red, blue, and green bars represent the environment-related features, those related to dimension reduction, angle-related features, magnitude-related features, and PA-related features, respectively. Each bar indicates the sum of the related features. For example, the angle-shift features are visualized from the sum of six features related to angle-shift, as described in Table 1. This result shows that not only PA-related features but also angle- and magnitude-related features are used in the prediction with GBR. In addition, a comparison between “pca-ang” and the angle-related features reveals that the dimension reduction reflecting the time-series data affected the importance of the features. By focusing on the result obtained for “magnitude-diff,” the feature importance of “mag-dd-diff” produced the highest result. This can be seen from the result shown in Figure 4A, in which the averaging of the light conditions did not represent the overall conditions, although that under dark conditions did, because it tended to show a higher rate of change in light conditions (e.g., about 0.02 to 0.08 for 120–140 h). This pattern can also be seen from “angle-diff,” in which the difference between “ang-ld-diff” and “ang-dd-diff” is small compared to that between “mag-ld-diff” and “mag-dd-diff.” This result can also be explained by Figure 4B, in which the change rate of angle in the light condition is higher than that in the dark condition. To further increase the accuracy of the model, SVR was used in Figure 5E. Using this model, the calculated correlation coefficient was 0.743.

## Discussion

Information and communication technology (ICT) has been used in the agricultural sector as a means of producing high-quality crops (Ibayashi et al., 2016). In the present study, the authors developed an MPI system, which uses microcomputers and a camera module to automatically collect time-series image data (Figure 1C). Compared to chlorophyll fluorescence, image data is easy to collect and analyze. Furthermore, the setup of the MPI system is convenient, which should lead to this technology being adopted by commercial plant factories. To extract those characteristics related to the circadian rhythm from this time-series image, the leaf movement of the plants was considered.

In the present study, the authors applied normal-vector analysis to extract the features from the MPI system. The normal vector shown in Figure 3A is relatively small, in that it represents the uniform elongation of individual leaves at night time. This is in good agreement with the results obtained by Miyagishima et al. (2014). On the other hand, Figure 3B shows a deviation in ***n***_ijk_, which results in a relatively large value of ***N***_k_ at day time. It is assumed that the increase in the angle defined in a previous study (Dornbusch et al., 2014) could be extracted from this result. It is obvious that the movement resulting from the elevation of the plant is greater than that resulting from the elongation, such that the results obtained for 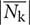 in Figure 4A are closer to the time-series pattern of the elevation angle rate rather than to the elongation rate reported by Dornbusch et al. (2014). The interpretation of the normal vector will be more difficult as the plant growth leads to the formation of multiple vectors. This issue can be resolved by isolating an individual leaf rather than by attempting to analyze an entire plant.

Figure 4A,B show the association between the LD cycles and 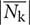 and θ_k_, respectively. This circadian rhythm can also be observed by calculating the 12-h moving average of the difference in *S*_k_ (Supplementary Figure S2). Although 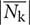 is able to extract circadian rhythms, the rhythms extracted from it are noisy relative to those taken from *S*_k_ The rhythm taken from θ_k_ also exhibits a complex feature, but a 24 h pattern and an indefinite change in the angle can be observed, which matches the characteristics of the plant phenotype. This complexity cannot be explained using existing plant science and knowledge of plant circadian rhythms, which is based on studies using simple waveforms. This should be discussed in the future from the viewpoint of plant morphology. The values of 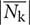 and θ_k_ are derived from complex data which are caused by a mixture of plant growth and elevation angle, as discussed before. To reduce the complexity of these data and reduce the amount of noise, it is necessary to extract the information derived from the circadian rhythm by averaging the light-on and light-off conditions. The “angle-shift,” “magnitude-shift,” “angle-diff,” and “magnitude-diff” features were applied to extract the features derived from the circadian rhythm. As can be seen from Figure 5D, the PCA features of the angle appeared low, as did the magnitude, relative to the PCA features of PA. It can say that the statistical analysis performs well for the simple results such as PA, but it is difficult to reduce the dimensions of data having a high degree of complexity, especially “ang-data.” Furthermore, the results of the dimension reduction, as derived from the circadian rhythms, exhibited a high “ang-data” value, relative to that of “pca-ang,” which leads to the conclusion that the simplification of complex data derived from the circadian rhythms provides a successful means of predicting plant growth. In addition, the result obtained for “mag-dd-diff” is as high as those for “PA-ave,” “PA-ld-diff,” and “PA-dd-diff,” such that it can be said that “mag” data would be an exceptionally reliable feature provided the dimensions were correctly reduced.

The SVR-based plant growth model produced a correlation coefficient of 0.743. This was an improvement over the former growth prediction model, thus pointing to the accuracy of the proposed model (Moriyuki and Fukuda, 2016). Furthermore, Guilford (1942) proposed that correlation coefficients of more than 0.70 be classed as a “High correlation; marked relationship,” again pointing to the quality of the proposed model. Furthermore, the proposed method proved capable of predicting the fresh weight at 22 days in the future with a high level of accuracy, whereas a former study only addressed that at 11 days in the future (Moriyuki and Fukuda, 2016). It is surprising that the prediction was successful, given that the experiment involved moving the plants from the nursery stage to cultivation stage prior to the measurement of the fresh weight, indicating that the prediction is possible without any knowledge of the environmental conditions in the cultivation stage. Although the evaluation using the proposed model with a correlation coefficient score proved successful, a few problems remain. Figure 5C,E show plots with a steep trend. This suggests that the model overestimates fresh weights that are somewhat lower (approximately 40–60 g) and underestimates those that are higher (approximately 100–120 g). These types of predictions are commonly observed in machine learning and are a result of there being an unequal number of data, as can be seen in the histogram of the fresh weight shown in Figure 5A. To overcome this issue, up- or down-sampling of the data can be performed, which is a machine learning process to duplicate the number of low-frequency observed data or reduce the number of high-frequency observed data. In addition, combining the prediction results obtained by multiple types of models, which is called ensemble learning, can improve the results in an actual commercial application, although in this work, simple models such as GBR and SVR were used to focus on both the prediction and description of the results.

The ability to predict plant growth prior to harvesting based on the feature values of lettuce seedlings at the nursery stage would greatly improve production stability in a plant factory. Moriyuki et al. (2018) investigated a profit model for a commercial plant factory. In their study, they focused on plant growth dynamics (the average and standard deviation of the fresh weight) as well as the shipping type, finding that the yield at harvest time was highly dependent on the growth dynamics of the lettuce population. In the present study, the authors devised a method of predicting the accuracy of the average and standard deviation of the fresh weight at harvesting by applying advanced analysis using the feature values of lettuce seedlings and machine learning, as shown in Figure 5E. Thus, the prediction of the yield at an early stage by applying an MPI system would lead to production stability in that the cultivation conditions could be adjusted once a predication had been made. It is suggested that the plant factory industry should implement such a means of prediction, whereby the necessary values are obtained using an MPI system and machine learning.

The present study displayed the capabilities of OF and normal-vector analysis when applied to a plant-growth prediction model. The results obtained from the models show the contribution of the features derived using OF and normal-vector analyses to machine learning. The results were obtained using an MPI system which uses only image data, and thus could easily be applied commercially.

## Author Contributions

HF and SN designed the research. SMperformed the experiments and data analysis with equal contributions to the first author. SN analyzed the data. HM and KW advised on the analysis methods. SN and HF wrote the manuscript. All the authors discussed the results and implications and commented on the manuscript.

## Conflict of Interest Statement

The authors declare that the research was conducted in the absence of any commercial or financial relationships that could be construed as a potential conflict of interest.
