# Supplementary figures and images for: Leaf-Movement-Based Growth Prediction Model Using Optical Flow Analysis and Machine Learning in Plant Factory
Source: Front Plant Sci. 2019 Mar 22;10:227. doi: 10.3389/fpls.2019.00227 (PMC6439531; doi:10.3389/fpls.2019.00227)

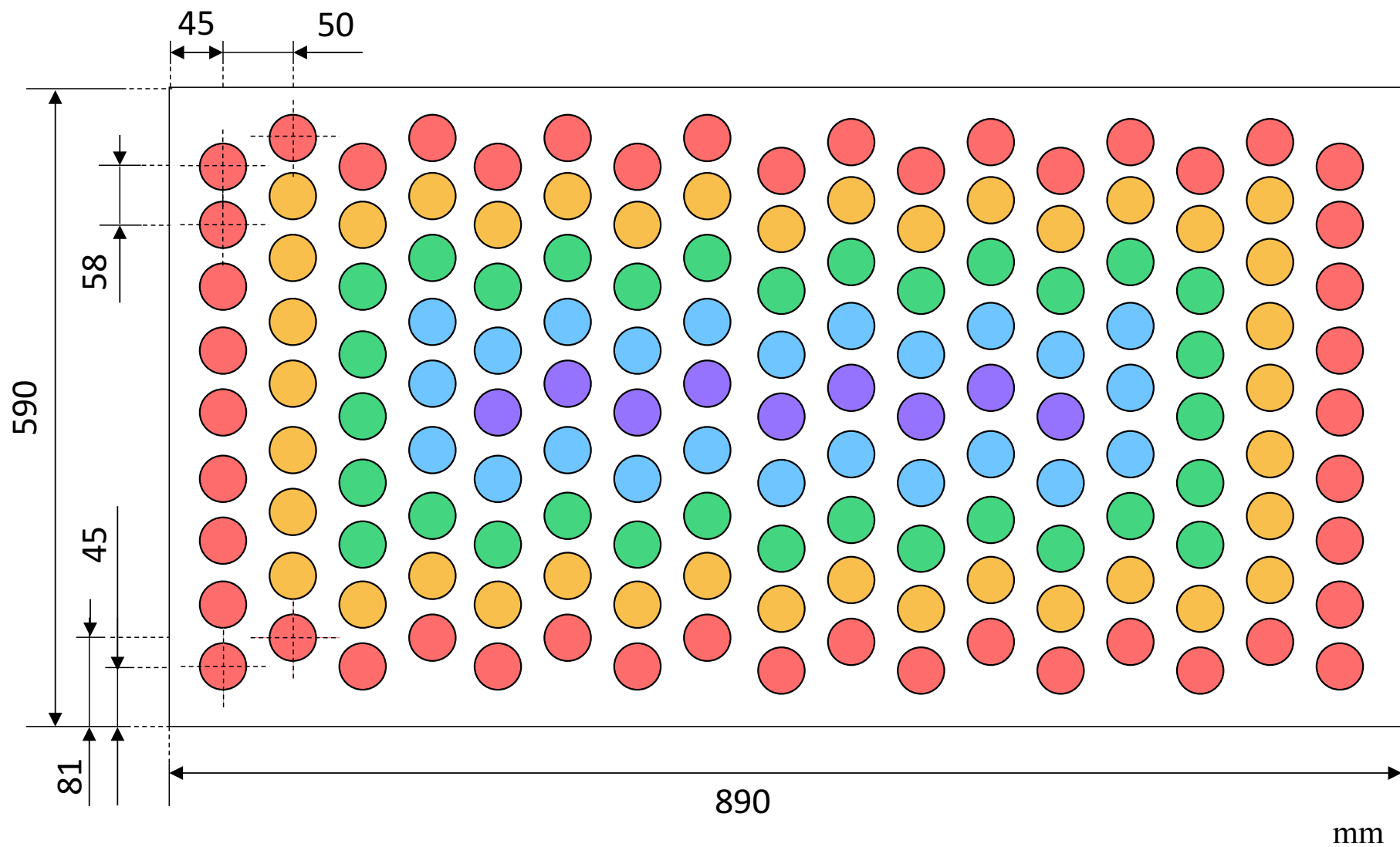

**Figure S1** Overview of the Feature 'Track'

Supplement: Supplementary file 1 [file Data_Sheet_1.PDF]
