# Supplementary material for: Leaf-Movement-Based Growth Prediction Model Using Optical Flow Analysis and Machine Learning in Plant Factory
Source: Front Plant Sci. 2019 Mar 22;10:227. doi: 10.3389/fpls.2019.00227 (PMC6439531; doi:10.3389/fpls.2019.00227)
Supplement: Supplementary file 2 [file Data_Sheet_2.PDF]

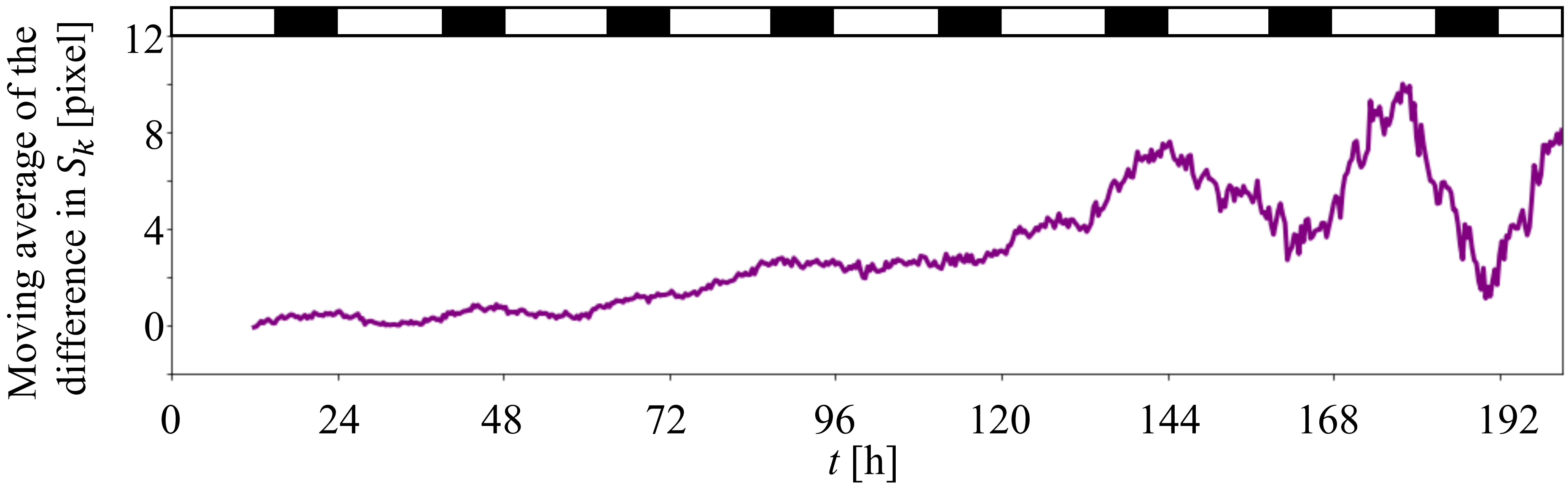

Figure S2 Moving average of the difference in S_k . The data were calculated by using S_k of Figure 4A. For the moving average, a simple moving average method was used. The window size of moving average was set to 12 h.
